# Low levels of classical BSE infectivity in rendered fat tissue

**DOI:** 10.1186/s13567-018-0618-7

**Published:** 2018-12-20

**Authors:** Christine Fast, Markus Keller, Martin Kaatz, Ute Ziegler, Martin H. Groschup

**Affiliations:** Institute of Novel and Emerging Infectious Diseases, Friedrich-Loeffler-Institute, Südufer 10, 17493 Greifswald-Isle of Riems, Germany

## Abstract

BSE infectivity in mesentery fat is most likely associated with embedded nervous tissue. To prove this mesentery containing celiac ganglion was taken from oral BSE infected cattle in different stages of the disease and from one control animal. Fat was rendered according to standard tallow production methods and the prion infectivity therein analysed in transgenic mouse bioassay. Rendered fat of the clinical animal revealed low infectivity levels, whereas preclinical and control animals remained negative. This study, although not representative, provides a proof of principle, indicating the potential contamination of melted mesenteric fat by embedded nervous structures during standard tallow production.

## Introduction, methods and results

Bovine spongiform encephalopathy (BSE) is a zoonotic [1], fatal neurodegenerative disease, belonging to the group of transmissible spongiform encephalopathies (TSE). All TSEs are associated with the accumulation of the pathological prion protein (PrP^Sc^), which results by the conversion of the normal host-encoded cellular prion protein and is the marker of the disease [2]. Among other TSEs classical BSE is transmitted via the oral uptake of infectious material [3, 4]. Besides cattle, cats [5], goats [6] and humans [1] are susceptible to the disease by oral uptake of infectious foodstuff. During the incubation period the BSE agent spreads from the gut, in particular the ileum, via sympathetic and parasympathetic nerve fibers and ganglia to spinal cord and brain stem [7, 8]. In orally BSE infected cattle first traces of infectivity can be detected in splanchnic nerves and in the celiac and mesenteric ganglion complex (CMGC) at 16 and 20 months post-infection (mpi), followed by an increase of infectivity until to the clinical stage when even a PrP^Sc^ accumulation can be seen in the ganglion [8]. Therefore, it is widely accepted that infectivity in mesentery, if present at all, is most probably associated with fat embedded nerve fibers and autonomic ganglia [9]. However, studies using fat from BSE affected cattle failed to detect any infectivity in RIII wild type mice [10]. More recently infectivity in adipose tissues has been shown for murine adapted scrapie and for chronic wasting disease (CWD) [11, 12].

The BSE exposure risk to humans and animals is currently minimized by the removal of specified risk material (SRM) from the food and feed chains. SRMs, as defined in Regulation (EC) No. 999/2001 as amended, are tissues containing the highest level of infectivity in BSE respectively TSE incubating animals. These include, among others, intestine and mesentery of animals of certain age/species from animals whose origin is in Member States or third countries with controlled or undetermined BSE risk. In this regard it has to be bear in mind that mesentery contains a network of nerves, ganglia, blood and lymph vessels as well as lymph nodes to support the gut [9]. However, up to now, it remains unclear whether a BSE contamination of rendered fat could occur during tallow production. Therefore, the study presented here intended to clarify, whether BSE infectivity can be found in fat rendered from mesentery fat embedded CMGCs of BSE infected cattle at different stages of the disease. Obtained data are important for qualitative and quantitative risk assessments and for the still ongoing discussion regarding the further usage of ruminant adipose tissue.

All infection experiments in cattle (LVL-MV 310-4/7221.3-1.1-019/02) and mice (LALLF 7221.3-1.1-25/09) described in this manuscript were approved by the competent authority of the Federal State of Mecklenburg-Western Pomerania, Germany, on the basis of national and European legislation, namely the directive 2010/63/EU on the protection of animals used for scientific purposes.

**Figure 1:**
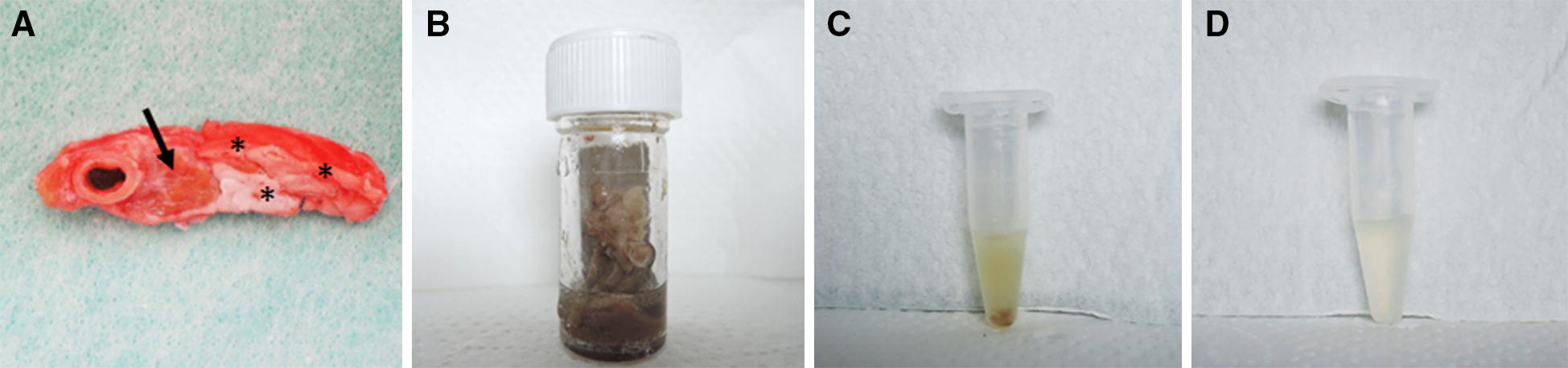
**Processing of the inoculum. A** Fat was rendered from the Celiac and mesenteric ganglion complex samples (arrow) which were embedded in mesentery fat (asterisk); **B** samples were incubated for 20 min at 95 °C according to standard tallow production methods; **C** the melted fat was taken, but still contained tissues remnants; **D** 100 µL of the liquid fat was 1:5 diluted in physiological saline, thoroughly vortexed and cleaned by a short centrifugation at 10 000 rpm. The resulting supernatant was taken as inoculum.

The CMGC samples were taken from four BSE infected cattle and one control animal (KT30, 20 mpi), all of them were part of the German BSE pathogenesis study [7, 8]. Two infected animals were at a preclinical time point (IT46, 16 mpi and IT60, 20 mpi), one animal at a late preclinical (mild PrP^Sc^ accumulation in the brain stem, but no clinical signs, IT61, 32 mpi) and one animals was at a clinical stage of disease (IT49, 36 mpi). For all cattle, mouse bioassay results for the CMGC were generated previously [8], showing either no, mild, moderate or substantial infectivity loads. Anamnestic data are given in Table 1. Fat was rendered from CMGC samples, which were embedded in mesentery fat, by incubation for 20 min at 95 °C, according to standard tallow production methods [9]. Subsequently the melted fat was taken, but still contained tissue remnants. Therefore, 100 µL of the liquid fat was diluted 1:5 in physiological saline, thoroughly vortexed and cleaned by a short centrifugation at 10 000 rpm. The resulting supernatant was taken as inoculum (Figure 1) to analyse the prion infectivity load by mouse bioassay using bovine PrP (Tgbov XV) overexpressing transgenic mice [13]. Depending on the sample size, 7–12 Tgbov XV mice were intracerebrally inoculated with 25–30 µL of the supernatant. During the incubation time all mice were assessed for the onset of clinical symptoms at least twice a week. Mice were sacrificed at the latest after 730 days or when showing clinical signs. Subsequently the mouse brains were examined for the accumulation of PrP^Sc^ by a discriminatory immunoblot using the C-terminal-monoclonal antibody (mab) L42 and the N-terminal antibody mab P4 [14]. Additionally, mouse brains were examined by immunohistochemistry, using the mab R145 in a dilution of 1:250 in goat serum. The pretreatment included an incubation with 98% formic acid followed by 3% H_2_O_2_/methanol and subsequent autoclaving in citrate buffer (pH 6.1).

**Table 1:** Anamnestic data of the celiac and mesenteric ganglion complex used and results of the mouse bioassay done with rendered mesentery fat

	Animal ID	mpi	Results CMGC [8]		Results rendered fat
			Mouse-bioassay (Tgbov XV)	IHC	Mouse-bioassay (Tgbov XV)
Negative control	KT30	20	Not done	Negative	0/11 > 629 dpi
Preclinical	IT46	16	+ (2/12)	Negative	0/10 > 664 dpi
	IT60	20	++ (7/14)	Negative	0/8 > 592 dpi
Late preclinical	IT61	32	++ (9/13)	+	0/7 > 633 dpi
Clinical	IT49	36	+++ (7/9)	+	1/6 586 dpi

mpi: months post-infection, CMGC: celiac and mesenteric ganglion complex, IHC: immunohistochemistry, *+* mild, *++* moderate, *+++* severe infectivity respectively accumulation of PrP^Sc^; the numbers in brackets shows the number of positive mice/inoculated mice, dpi: days post-infection.

**Figure 2:**
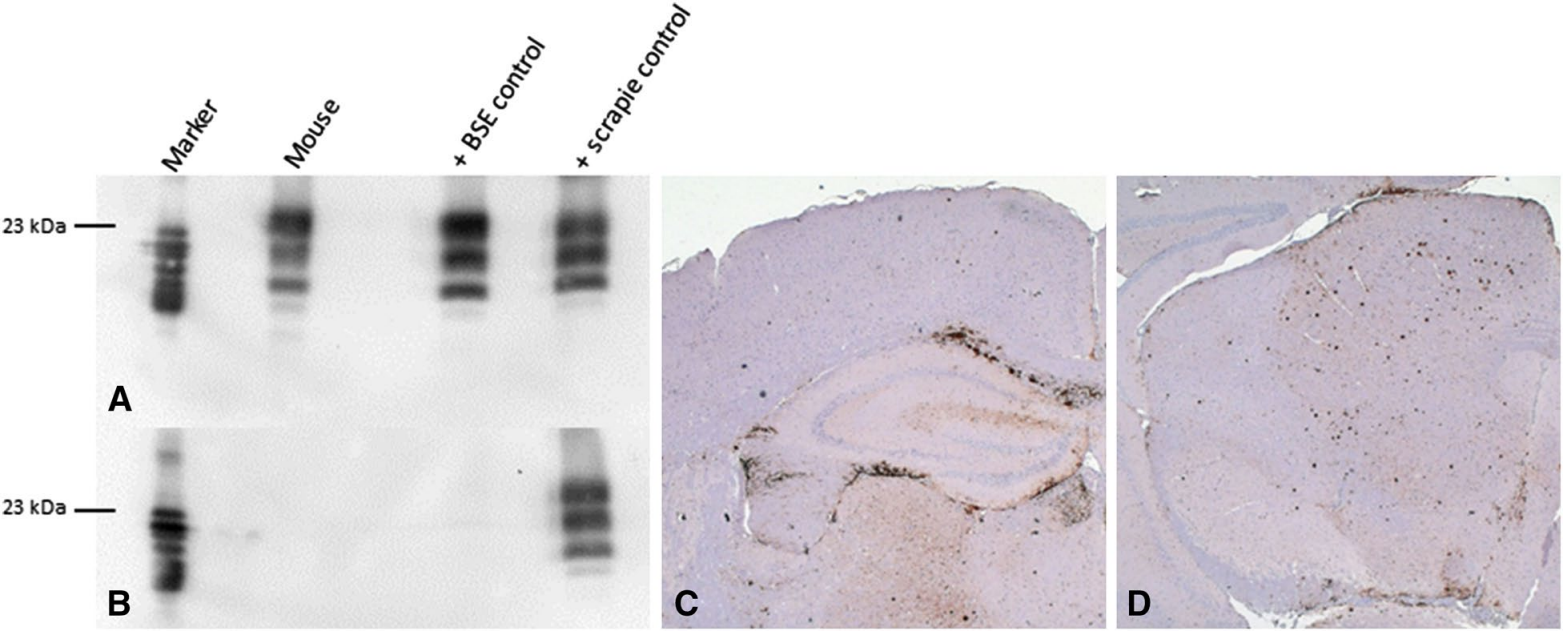
**Discriminatory immunoblot and IHC of positive mouse inoculated with rendered fat.** Inocula was prepared from the CMGC of the clinical cow IT49, 36 mpi. Typical for BSE (**A**) C-terminal antibody mab L42 shows a clear signal of PrP^Sc^ but not (**B**) N-terminal antibody mab P4, only the scrapie control can be detected. Additionally the same brain reveals clear plaque-like PrP^Sc^ accumulation in particular in the Corpus Callosum (**C**) and mid brain (**D**), immunohistochemistry, mab R145.

Results of the mouse bioassay are shown in Table 1. Neither the control and the preclinical nor the late preclinical animals showed signs of infectivity in mouse bioassay of the rendered fat samples after up to 730 days post-infection (dpi). In contrast, low levels of infectivity were detected in the fat of the clinical animal (IT 49, 36 mpi) as one mouse displayed a clear accumulation of pathological prion protein in the brain after an incubation period of 586 dpi (Figure 2). As expected for BSE a clear PrP^Sc^ deposition was observed in the discriminatory immunoblot using a C-terminal antibody (mab L42, epitope 145–163), but not with an N-terminal antibody (mab P4, epitope shp 89–104). The same mouse brain displayed in IHC a clear plaque-like accumulation of PrP^Sc^ in particular in corpus callosum and in the mid brain.

## Discussion

The results presented here indicate that a certain amount of BSE infectivity must be present in the mesentery to contaminate the rendered fat in a detectable level during tallow production methods. As shown by [8] BSE infectivity increases in the CMGC of orally BSE infected cattle from early to late preclinical up to the highest levels at clinical stage. Hence, it comes without surprise that the infectious fat presented here originates from the CMGC of the clinical animal, which also showed a clear PrP^Sc^ accumulation pattern [8]. Additionally these results are in accordance with the infectivity data reported for fat tissue of CWD infected deer at later stages of the disease [12].

In our hands only low level of infectivity were found. However, the experimental adipose/CMGC tissue dilution factor needed for the fat rendering should also be considered, so the actual infectious load of the samples might be higher. However, the low infectivity load found here might explain why earlier studies failed to detect BSE infectivity in adipose tissue. For one reason this studies did not definitely regard nerves and ganglia which are involved in the pathogenesis of BSE and for another reason less susceptible conventional wild type mice were used [10, 15, 16]. Interestingly omental fat of deer infected with CWD at a clinical stage revealed a much higher infectivity level as compared to our results [12]. Therefore, it is tempting to speculate that these differences might be due to the qualitative and quantitative more widespread CWD distribution in bodily tissues [17] as compared to cattle BSE. However, it has to bear in mind that the two TSE strains are different, different mouse line were used and neither for CWD samples nor for our BSE sample a calibration curve for infectivity exist. Nevertheless, it would be of interest to what extent rendered fat could be contaminated by using mesentery from sheep and goats infected with classical scrapie or BSE, all entities showing in most cases a higher PrP^Sc^ accumulation in the autonomous nervous system than BSE infected cattle [18–21].

Our results, in particular the close relationship with the infectivity/PrP^Sc^ data of the CMGC samples, clearly support the widespread accepted assumption that infectivity in the mesentery is most probably associated with nerves and autonomous ganglia [9], whereas the direct involvement of fatty cells is uncertain [11]. Another source of infectivity could be in mesentery fat embedded lymph nodes. However, no infectivity has ever been detected in mesenterial lymph nodes of BSE infected cattle so far [13, 22] and all grossly evident lymphoreticular tissue was removed from our samples. Additionally a long term study showed that full blood transfusion from clinical BSE infected cattle to naïve calves did not transmit BSE [23]. Therefore, a blood contamination of the sample as possible source of infectivity is highly unlikely. Furthermore, as all tissue remnants in the rendered fat were removed by a centrifugation step before inoculation, all infectivity must be bound to the liquid fat solely.

These results might not be representative due to the small sample size and therefore provide only a proof of principle. In particular with regard to the negative early and late preclinical samples, there still remain some uncertainties, which can only be resolved by a more extended, that say statistical profound study. However, the inocula were generated according to standard tallow production methods [9], therefore our results clearly show that such a contamination is conceivable. Rendered fat can been used for food (i.e. premier jus, frying agent), pet food and feed application [24], therefore BSE/TSE infectivity could enter both the food and feed chain. At the time of writing the current SRM legislation prevented the usage of mesentery fat from animals whose origin is from countries with controlled or undetermined BSE risk. However, this regulation is still under discussion and might be changed in near future (EU Commission, personal communication).

## Abbreviations

BSE: bovine spongiform encephalopathy
TSE: transmissible spongiform encephalopathies
PrP^Sc^: pathological prion protein
CMGC: celiac and mesenteric ganglion complex
CWD: chronic wasting disease
SRM: specified risk material
mpi: months post-infection
mab: monocloncal antibody
